# Influence of Short-Term Aging on Mechanical Properties and Morphology of Polymer-Modified Bitumen with Recycled Plastics from Waste Materials

**DOI:** 10.3390/polym12091985

**Published:** 2020-08-31

**Authors:** Clara Celauro, Edwina Saroufim, Maria Chiara Mistretta, Francesco Paolo La Mantia

**Affiliations:** 1 Dipartimento di Ingegneria, Università degli studi di Palermo, 90100 Palermo, Italy; mariachiara.mistretta@unipa.it (M.C.M.); francescopaolo.lamantia@unipa.it (F.P.L.M.); 2 Department of Civil and Environmental Engineering, Faculty of Engineering, University of Balamand, Koura 1300, Lebanon; edwina.saroufim@fty.balamand.edu.lb

**Keywords:** polymer-modified bitumen, waste polymers, short term aging, production temperature, mechanical properties, morphology, microscopy

## Abstract

Polymer-modified bitumen (PMB) is bitumen that has been specifically engineered with polymer for providing enhanced performance in service. The aging of bitumen is a main aspect that is able to affect its final performance: during the production phase in a hot mix plant, all the binders experience short-term aging due to the high processing temperature. The same is true during the production of the modified binder, when the polymer is dispersed at high temperature in the bitumen mass. This paper aims at studying the effect of short-term aging when using different types of modifiers such as recycled polymers obtained from waste materials. A 50/70 penetration-grade bitumen has been modified, and bitumen characterization has been carried out before and after short-term aging; conventional tests, viscosity measurements, and dynamical mechanical analysis have been used to investigate the properties. Different aging indices have been determined for predicting the effect of short-term aging based on the type of modifier. Furthermore, the morphology of the modified bitumen has been investigated via fluorescent microscopy, before and after aging, in order to highlight morphological changes due to aging. The results confirm that aging affects all the modified binders, due to the thermal stress imposed during PMBs production. Nevertheless, polymer modification is proved to reduce the aging effect in terms of an increase in the elastic modulus as experienced by the original binder.

## 1. Introduction

In order to meet the increasing requirements for bituminous road pavements, mechanical resistance, and durability—mainly due to the increased traffic volumes and loads—since the early 1990s, the polymer modification of bituminous binders has been challenging road technologists as well as polymer researchers [1,2,3]. In fact, the modification of road bitumen with selected polymers enhances the performance of both the binder (polymer-modified bitumen, PMB) and the mixture produced, with a beneficial effect to the service life of the pavement. Nowadays, the use of PMB is explicitly required in many technical specifications for paving and maintenance application for highways and other roads belonging to the primary network, where high performance is needed.

PMBs are industrially produced by mechanical dispersion via the high shear mixing of a selected (virgin) polymer into the neat bitumen at a high temperature in a molten state. This modification is called “wet” modification, while another option for polymer modification is the “dry” one, where the polymer is added at the bituminous mixtures directly in the mixing chamber in an asphalt plant [4,5]. A typical problem encountered in PMB production is the compatibility between polymer and bitumen and the consequent stability of the modified binder after cooling and for prolonged times without stirring (storage stability), with the risks of macroscopic phase separation between polymer and bitumen. Furthermore, the production temperature of the PMB affects the premature aging of the base binder, so that the original properties of it are indeed changed due to both polymer modification and aging for production at high temperature.

The aging of bituminous binder (including PMBs) once in the mixture is typically considered at two different stages, the so-called short-term aging and long-term aging [6,7], but very few studies focused so far on the aging mechanism of the PMBs at the production stage. The short-term aging of bituminous binders is mainly due to the volatilization of the lighter components during the production of mixtures at high temperature in an asphalt plant and during laying operation on site. Long-term aging is referred to as that related to heat, air, and sunlight exposure of the mixtures once laid on site and in service in the pavement. Therefore, reduction in bituminous mixture’s durability is related to excessive hardening of the binder due to composition-related factors identified by Petersen [8] as follows:

- Loss of light components by volatility or absorption by porous aggregates;
- Changes in the chemical composition due to oxidation;
- Molecular structuring at low temperature and consequent thixotropic effects (time-dependent, steric hardening).

Volatilization and oxidation are related to irreversible bitumen chemical changes, which are known as age hardening, while the reorganization of bitumen molecules is a potentially reversible phenomenon, which is also called physical hardening [9,10,11]

For PMBs, depending on the chemically active nature of the polymer dispersed, polymer degradation may also occur; thus, the combined effect of bitumen oxidation and polymer degradation, which varied with bitumen source/grade and polymer type/content, should be considered when studying the aging behavior of PMBs [1,12,13,14].

Therefore, the choice of an appropriate “aging index” is of paramount importance for polymer-modified asphalt, since artificial short-term aging procedures are not specifically designed for modified binders. Therefore, in order to evaluate the aging performances, it is preferable to compare and complement information obtained by more aging indexes, in order to avoid any possible misinterpretation of artificial aging results.

This study aims to evaluate both the morphological characteristics and mechanical performances after the standard short-term aging of several PMBs through the rolling thin film oven test (RTFOT) according to standard EN 12607-1, produced in the laboratory with different types of waste or treated polymers, with the same base bitumen. A correlation between the effects of aging on the morphology of the polymer-modified bitumen is discussed in relation to aging indices obtained after short-term aging via a conventional test (penetration, softening point), viscosity test, and dynamic tests carried out on the modified bitumen produced.

## 2. Materials and Methods

### 2.1. Materials and Sample Preparation

Conventional bitumen having a 50/70 penetration grade with representative parameters shown in Table 1 was used as the base material for the modification of the blends studied in this paper.

Bitumen components (asphaltenes and maltenes, with the latter further divided into saturates, polar aromatics or resins, and naphthene aromatics) were determined following the IP 469-01 standard with the thin layer chromatography-flame ionization detector IATROSCAN MK6 TLC-FID/FPD analyzer. The results obtained are provided in Table 2.

Based on the previous results, according to the classical colloidal model for bitumen (considered as a colloidal suspension where asphaltenes are dispersed in maltenes and resins act as a peptizing agent for asphaltene micelles, while aromatics and saturates create an intermicellar continuous phase), it is possible to calculate the colloidal index (*CI*), representing the ratio of the flocculated fractions to dispersed ones, as follows [2,15]:

(1) $$CI=\frac{X_{ASPHALTENES}\;+\;\;X_{SATURATES}\;}{X_{AROMATICS\;}\;+\;\;X_{RESINS}}=0.234$$

It is interesting to note that the typical *CI* range for common types of bitumen is between 0.5 and 2.7 and that binders having *CI* < 0.7 exhibit typical sol-type behavior. Therefore, the *CI* of this neat bitumen (close to 0.3) proves to be highly suitable for the production of polymer-modified bitumen [15].

The polymers used in this work are reported in Table 3 together with some characteristics. The selection was made to explore the effect of a variety of types of polymers, with different chemical structure, morphology, and density range. All the virgin polymers have been processed by extrusion in a twin-screw extruder OMC (Officine Meccaniche Conte, Fondi, Latina, Italy) at a die temperature of 180 °C and reprocessed in a single screw extruder in order to simulate a recycling operation. Then, the polymer samples can be considered as a post-consumer polymer. After extrusion, the polymers have been cut in a granulator to allow dispersion into the bitumen.

Moreover, the same sample of LDPE has been used in an LDPE-LLDPE-EVA (LD/LL/EVA) ternary blend whose composition is 75/5/20 wt % and is representative of the average composition of typical films for greenhouses. In addition, this blend has been prepared by extrusion in a twin-screw extruder OMC at a die temperature of 180 °C and the extruded material was granulated in small round pieces. This ternary blend aims to improve the compatibility of the polyolefin with the bitumen through the addition of polar groups (EVA) [16,17]. Thus, the EVA was selected with a vinyl acetate content that ensures a balanced degree of crystallinity and polarity of the chains for compatibility with bitumen [18].

For modification purposes, also an elastomeric modifier, crumb rubber modifier (CRM) from discarded tires, was used. The potential use of a crumb rubber as a bitumen modifier has been extensively studied due to its technical and environmental benefits: the effect of rubber modification is comparable to that of styrene-butadiene-styrene modification, but it can provide a significant cost saving due to the high price of virgin SBS together with the prevention of accumulation of this waste material in landfill [19,20]. Recently, some authors also focused on the effect of aging on the wet blend (crumb rubber dispersed in bitumen) as well as on the role of thermodynamics and kinetics in rubber–bitumen systems [21,22].

The waste CRM was supplied by a tire recovery plant: it is obtained via mechanical grinding at air temperature. Due to the high heterogeneity of this material, whose composition varies depending on the variable proportions of natural, synthetic rubber and other components between truck and passenger car tires, no chemical test was carried out, considering the low representativeness of the samples for chemical characterization, with respect to the overall quantity of the waste material to be used.

Therefore, attention was paid to the average and qualitative characteristics of the materials, which are detailed in Table 4.

**Figure 1:**
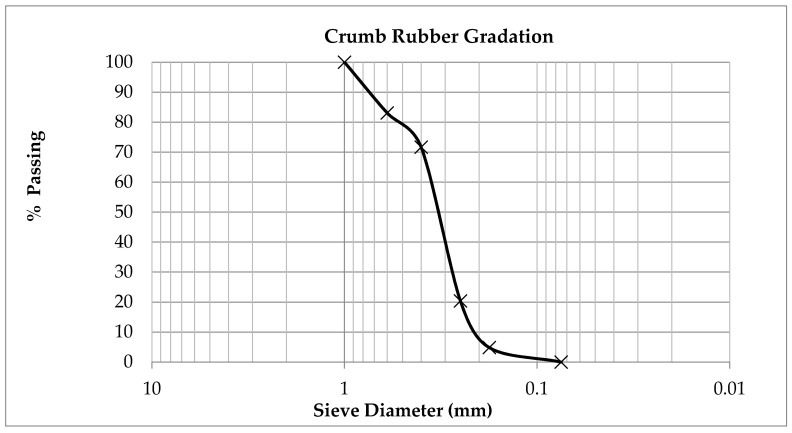
Gradation of the crumb rubber modifier (CRM) used.

Special attention was paid to CRM gradation: as regards the maximum size of its elements, this was limited to 1 mm to improve the dispersion as well as the reaction with bitumen. More than 70% of the rubber used has a dimension less than 0.4 mm, as can be seen in Figure 1.

For comparison purposes, all the modified bitumen studied were produced by adding the same percentage of each selected polymer (see Table 3) to the base bitumen. Regardless of the type of polymer, 5 wt % was selected as in previous studies [17] for all the blends produced to guarantee significant modifications to the mechanical, rheological, and morphological properties of the neat bitumen; thus, it is not a content optimized for specific production or usage needs (storage at high temperature or elastic behavior). The modified binders were obtained by mechanical dispersion of the polymers in the base bitumen under high shear. Bitumen was heated to 180 °C in the oven and then transferred into a double-wall thermostatic bath. Then, 5 wt % of each polymer was added into bitumen, and the mixture was blended in a high shear mixer for 2 h after all the quantity was fed in at a constant shear of 4000 rpm. After mixing, the blends produced were visually inspected in order to macroscopically detect the potentially segregated phase of the dispersed polymer.

In addition, the neat bitumen was subject to the same mixing protocol, without any polymer, in order to investigate the aging effect of the process when preparing modified mixtures. This thermally stressed binder, hereafter named “Bitumen 50/70 mixed”, was subject to dynamic viscosity and dynamic mechanical analysis, for comparison purposed with the polymer-modified binders produced.

### 2.2. Aging Procedures and Testing Methods

In order to simulate the aging effect that occurs during the production and paving operations of hot mix asphalt, i.e., short-term aging, the EN 12607-1 standard procedure was followed, using the rolling thin film oven test (RTFOT) at a constant temperature of 163 °C (the sample is subject to oxidation by a 4000 mL/min air flow and a rotation rate of 15 rpm for 75 min).

A conventional penetration test and softening point tests were performed according to EN 1426 and EN 1427, respectively, before and after aging. Storage stability with the so-called “tube test” and elastic recovery in a ductilometer at the testing temperature of 25 °C, according to EN 13399 and EN 13398, were carried out, too, since they represent specific requirements for the acceptance of PMBs for road pavement construction.

All the modified binders produced were also tested for rotational viscosity from 100 °C to 180 °C at both the aged and unaged stage. In fact, a rotational viscometer is used to measure the viscosity of the material as an indicator of its flowing behavior at high temperatures. Thus, it is performed on unaged asphalt binders to determine the flow physical characteristics of the material that are of interest in asphalt plant, for mixture production at high temperature. The equipment used to measure the viscosity is the Brookfield apparatus, DVIII™ ultra Rheometer, and its compatible Thermosel System to control sample temperature.

Furthermore, according to EN 14770, isothermal frequency sweeps tests under stress-controlled mode were carried out with an Anton Paar Physica MCR 101 dynamic shear rheometer (DSR) to investigate the rheological properties of the bitumen in the linear viscoelastic (LVE) range (applied strain below the limit of the LVE region) as determined via amplitude sweep tests with a parallel plate geometry. The maximum applied strain for the frequency sweep tests was 1%. Two plate geometries (8 mm and 25 mm) were used, according to the binder viscosity, in order to cover the wide testing temperatures ranging from −10 to 80 °C. The rheological properties of the binders were measured in terms of their viscoelastic functions: complex (shear) modulus G*, storage modulus, G′, loss modulus, G”, and phase angle δ. A minimum of two replicates was carried out per each binder studied.

The morphology of polymer bitumen blends was carried out too, according to the method designated in the European Standard, EN 13632, via standardized attribution to the blend of a letter coding format related to the nature of the continuous phase as well as to the description of the phase, size, and shape of the dispersed material, according to the letter coding description detailed in Table 5. In this method, the PMBs are illuminated under ultraviolet (UV) light in a microscope, and the polymer was dispersed in the bituminous matrix fluoresces. Thus, it is possible to qualitatively describe the morphology of the modified binders. Specific samples preparation is prescribed in order to preserve the instantaneous morphology: this procedure includes pouring each modified binder in steel plates (preheated at the same bitumen temperature) and cooling them at room temperature for a couple of hours. Then, samples are stored at low temperatures where the brittle properties of bitumen are achieved. Afterward, the polymer–bitumen specimens are fractured with a sharp tool in order to have a regular surface and placed for investigation under the microscope. The images for this study were taken with a 16:1 magnification using a trinocular OPTIKA^®^ microscope, N-400FL model.

## 3. Results and Discussion

### 3.1. Conventional Tests

The results obtained from the conventional test of penetration (that simulates the binder consistency at intermediate temperature) and softening point (that simulate the consistency at high temperatures) are summarized in Table 6.

The same table provides the Penetration Index, PI, calculated for neat bitumen and modified binders, based on the softening point results and standard penetration at 25 °C [23] that is conventionally assumed as an indicator of the thermal susceptibility of bitumen: the lower the PI, the higher the susceptibility. For use in road pavements, a PI between −1 and +1 is generally associated to suitability for road construction. In this range, according to the previously mentioned colloidal model, the bitumen structure is the sol–gel type, which is an intermediate condition between the proper sol type (asphaltenes well dispersed and non-interacting, PI < 0) and the gel-like structure (when asphaltene micelles show a strong agglomeration tendency, PI > 2) [24].

From Table 6, it is possible to notice a decrease in the penetration for the unaged binders: for the same polymer content, the higher the density of the polymer, the higher the reduction. On the contrary, the softening point increases with polymer addition. As expected, there is an increase in bitumen stiffening (or hardening), and this effect is enhanced when the short-term aging procedure is carried out. On the other hand, the increase in softening point is favorable, since bitumen with a higher softening point may be less susceptible to permanent deformation (rutting). In any case, after polymer addition, all the polymer-modified bitumen maintain the sol–gel nature of the base bitumen, before and after aging (PI within the range −1.00 and 1.00), with a general decrease in thermal susceptibility (associated to an increase in the PI), except for the one modified with LD/LL/EVA.

In addition, Table 6 also shows the loss of light fractions due to exposure at high temperature during the RTFOT, which is calculated in terms of percentage of the initial mass of the sample. This provides a quantitative measure of the loss in volatiles that is related to an increase in binder viscosity. This loss is not significant for age-hardening evaluation, since age hardening is more related to oxidation phenomena than to volatilization that mostly affects rheological properties, due to the reduction of the dispersed fractions, resulting in a more aggregated “structure”.

According to the harmonized European standard EN 14023, for use in road pavement, a modified binder should have a drop in softening point—after RTFOT—∆T_R&B_ ≤ 5 °C, while the retained penetration should be ≥ 60% of the unaged one and the change in mass ≤ 0.5%. As seen in Table 2, at the selected polymer content, these requirements are fulfilled for the binder with LDPE-LLDPE-EVA processed, while the other blends do not satisfy all the requirements at the same time.

Table 7 provides results of the storage stability test, according to EN 13399, carried out for the modified binders produced: these results provide an indication of the heterogeneity of the bitumen/polymer blend when stored at high temperature, as it happens in a typical hot mix plant.

The same table provides the results of the elastic recovery test, according to EN 13398. For use in a hot mix plant, the PMB’s requirement are ΔT_R&B_ ≤ 5 °C and ER > 50%. As stated earlier, the blend was not optimized in terms of polymer content concerning these requirements; nevertheless, they confirm what was expected from the introduction of polar groups for improving compatibility with bitumen: the ternary blend LD/LL/EVA proves to be more stable due to the introduction of EVA but not sufficiently elastic for road requirements. For the CRM, the results suggest the opposite: adequate stability is not reached, but a satisfactory elastic recovery is obtained.

### 3.2. Dynamic Viscosity

Dynamic viscosity from 100 to 180 °C was determined for all the binders studied, at both the aged and unaged stage. The neat bitumen was also tested, for comparison purposes, after thermal stress simulating the one applied during PBM production (bitumen 50/70 mixed). The results are provided in Table 8.

As expected, because of the higher intermolecular associative interactions among polar oxygen-containing functional groups, which increase during oxidative aging, an increase of the viscosity is reported for all the binder studies, included the one that was subject to thermal treatment.

As it can be noticed, the thermal treatment of the base bitumen proves to have an effect in the increase in viscosity at both the unaged stage (neat versus mixed pre-RFTOT) and short-term aged state (mixed versus neat post-RFTOT): this proves that—to some extent—the increase in viscosity of the PMBs compared to the neat bitumen in both stages cannot be attributed to the presence of the polymer only, but also to the high temperature process related to PMB production. For the modified binders, it can also be noticed that the increase in viscosity after short-term aging is quite limited for the ones with the processed LDPE and with a ternary blend of LD/LL/EVA, while it is much higher for that with CRM.

The relationship between the viscosity, *η*, and temperature, *T*, can be modeled using the Arrhenius equation [25]:

(2) $$\eta \;=Ae^{\frac{E_{a}}{RT}}.\;$$

where *R* is the universal gas constant (8.314 J/(mol K)), *T* is the absolute temperature, *K*, *Ea* is the activation energy, J/mol, and *A* is the Arrhenius constant (or frequency factor constant).

**Figure 2:**
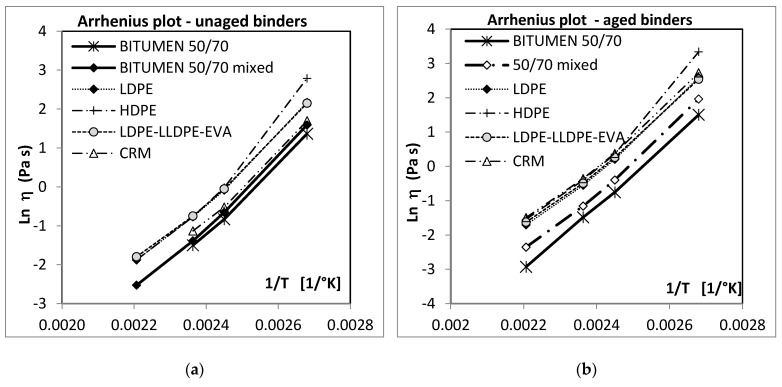
Logarithm of dynamic viscosity against the reciprocal of absolute temperature (Arrhenius plot) for the binder studied: (**a**) before aging; (**b**) after aging at the RTFOT.

A plot of ln(*η*) versus (1/*T*) is given in Figure 2a,b, for all the binders studied at the two conditions, unaged and aged, respectively. From the regression parameters of the straight lines obtained, the activation energy *Ea* can be determined. The results are provided in Table 9.

**Figure 3:**
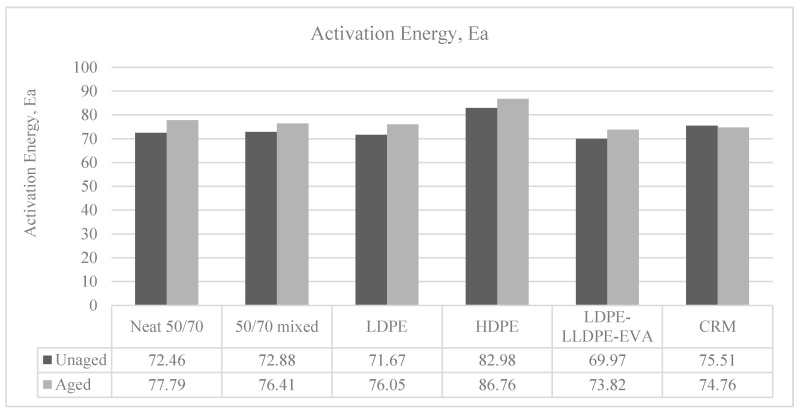
Effect of polymer introduction on the activation energy, pre and post aging.

As in other studies [11,26,27], for a selected binder, the activation energy increases with aging: this reflect the changes in binders’ components and molecular structures. The results are depicted in Figure 3. An increase in *Ea* corresponds to a worsening in the temperature sensitivity, since it is related to the energy needed to flow: after aging, this is to be attributed to the increase in the intermolecular forces due to oxidation.

In absolute value, the largest increase in the activation energy due to aging can be noticed for the neat bitumen, while polymer introduction seems to reduce this effect, and the binder with CRM shows a value almost constant even after short-term aging.

The effect of polymer modification of the same base bitumen leads to different results: on one hand, the activation energies of the PMBs with HDPE and CRM are higher than that of the neat bitumen at the unaged condition: at the aged condition, only that for HDPE is higher, while the one for CRM ends up being almost the same as that of the aged neat bitumen (this suggests that the 5% of CRM is close or above the critical polymer concentration beyond which the activation energy for flow decreases [26]. For LDPE and even more for the ternary blend LD/LL/EVA, a reduction in the activation energies can be noticed with comparison to the base binder, both at the unaged and the aged condition.

This variability in the changes in *Ea*, due to polymer modification, confirms that the different polymer types influence the interaction between the base binder and polymer dispersed into it.

### 3.3. Aging Indices

Based on the data provided in Table 6 and Table 8, further evaluation of short-term aging can be carried out through some classical aging indices [28], which are defined as:

(3) $$AI=\frac{X_{AGED}}{X_{UNAGED}}.$$

Table 10 details the viscosity aging index (RV), as well as for that Ring and ball temperature and penetration (PV). These parameters are assumed as indicators of the aging effect: higher sensitivity to aging is associated with higher values for the parameters ATR&B and ARV and lower values for the parameter APEN, whereas the opposite trend reflects lower sensitivity to aging [29].

As it can be noticed, there is no clear correlation between the information provided by the different aging indices AI_RV_, AI_TR&B_, and AI_PEN_ (%), and no specific trend can be detected for ranking aging effect on the binders. The discrepancies between these indices prove that they are not adequate for providing clear information about the aging effect on PMBs and that different types of indices have to be considered.

### 3.4. DSR Results

Analysis of dynamic data obtained in frequency sweep tests allowed one to conclude that in general, the time–temperature superposition principle holds for all the investigated materials; thus, all the master curves of the viscoelastic functions (storage modulus, loss modulus, and phase lag master curves) were constructed using the same horizontal shifting factor according to the Williams–Landel–Ferry Equation [30]:

log (*α_T_*) = −*C*_1_(*T* − *T_REF_*)/[*C*_2_ + (*T* − *T_REF_*)] (4)

where *T_REF_* is the reference temperature for master curve calculations and *C*_1_ and *C*_2_ are the material’s constants.

For comparison purposes, DSR tests were carried out on the neat bitumen after mixing at high temperature in the high shear mixer for PMBs production for simulating the effect of aging due to the mixing process. After horizontally shifting the isothermal curves obtained by the frequency sweep tests, smooth master curves of the viscoelastic functions were constructed for all the materials studied at a reference temperature of 30 °C.

As reported by some authors [13,29,30], master curves of the complex modulus, G*, as a function of the frequency, shift toward lower values of the frequency or show higher values at lower values of the frequency after aging. This feature means that the mechanical behavior of the material becomes more rigid because its relaxation is much lower at higher values. This behavior can be considered as a sort of liquid–solid transition or a viscous–elastic transition. It is worth recalling that a liquid relaxes instantaneously while a solid does not relax. The index of aging has been evaluated as the difference between the area of the aged sample and the area of the unaged sample.

Coherently with the previous analysis for the Storage Modulus master curve, the Rheological Aging Index (RAI) originally proposed by Cavalli et al. [31] and used in other studies [32], which is based on the difference between the area under the aged and unaged Complex modulus master curves within a definite range of reduced frequencies, is here modified and calculated for G′ master curves, in a double logarithmic scale:

(5) $$\mathrm{RAI}=\;\int_{0.001}^{1000}\log G\prime \left({\bar{\xi }}_{aged}\right)-\log G\prime \left({\bar{\xi }}_{unaged}\right)d\xi .$$

For comparison purposes, the limits of the integral have been chosen as the widest frequency limits in the G′ master curves, which are common to all the binders tested.

**Figure 4:**
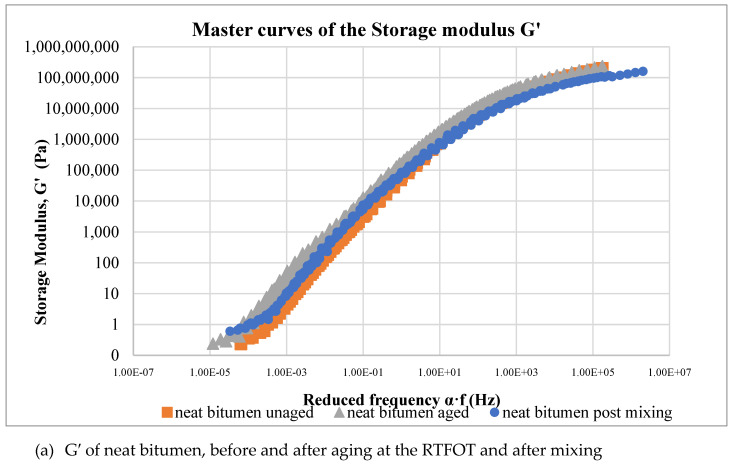

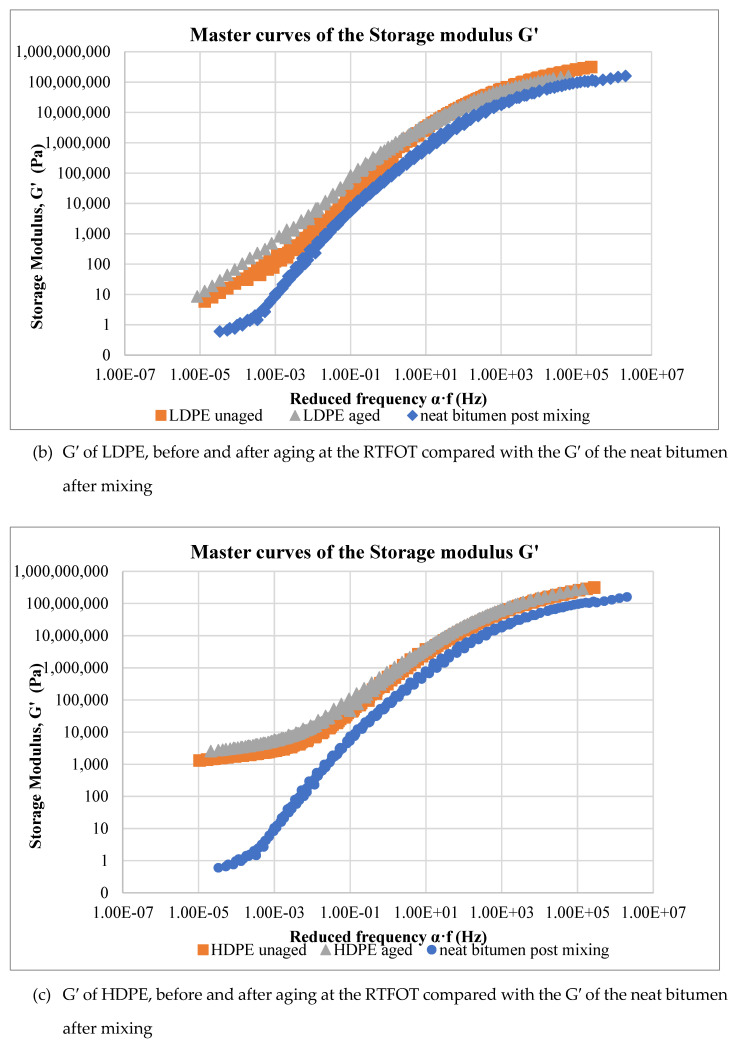

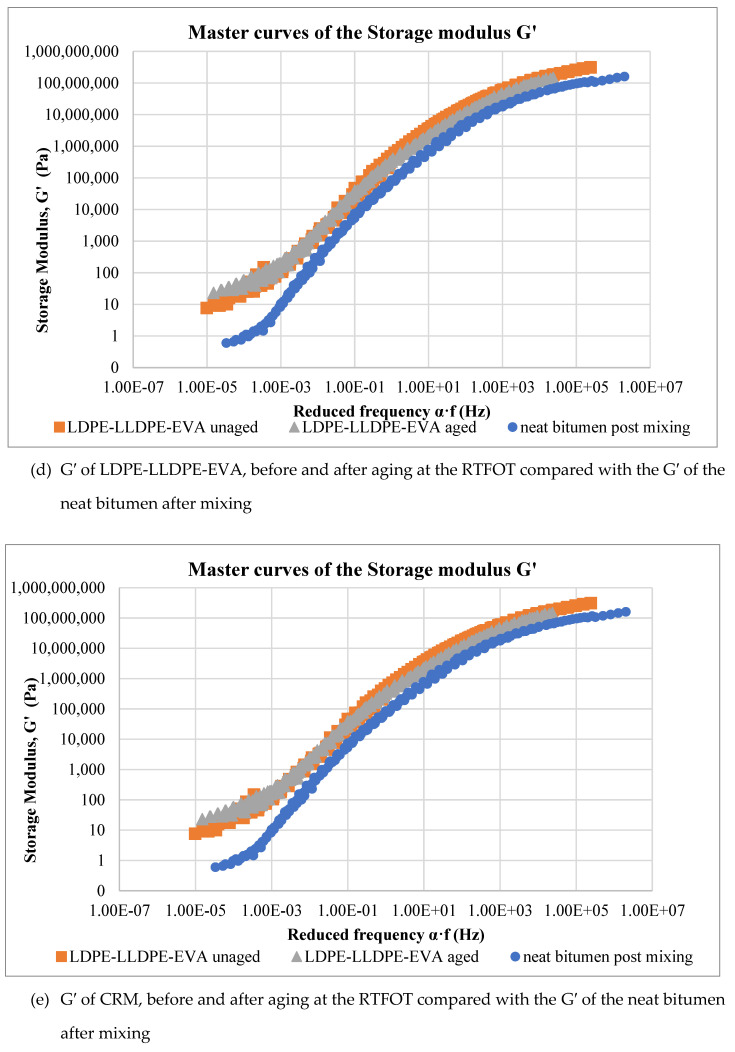
Master curves of G′ at 30°C for each polymer-modified bitumen (PMB) at aged and unaged conditions.

In Figure 4a–e, the values of the G′ modulus of both aged and unaged samples have been reported for all the investigated materials, as well as the one for the neat bitumen post mixing, for comparison purposes. The use of storage modulus, G′, instead of the G* modulus, is to be preferred as the storage modulus is just the measure of the elastic component of the material, and an increase of G′ with a change of slope of the G′–frequency curve is a measure of the viscous–elastic transition of the bitumen.

Both the curve of the unaged bitumen and of the modified samples shift toward lower frequencies but at different levels. The change of the G′ curve is remarkable for the unmodified bitumen, while this change is significantly lower for the modified bitumen. Moreover, the curves of the aged samples show at the lowest frequency a slope lower than that of the slope of the unaged sample. As reported before, a decrease of the slope in the lower frequency range is again a measure of the transition to a solid state as the slope of the solid materials goes to zero. Therefore, the effect of the polymer fillers is to slow the aging effects of the bitumen.

**Figure 5:**
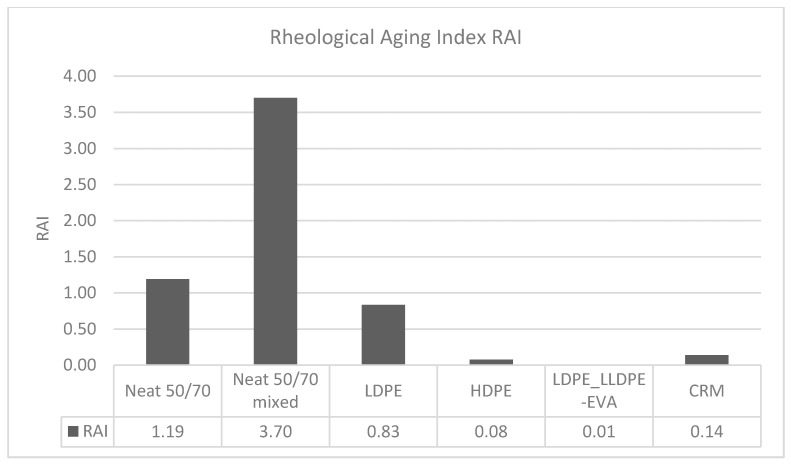
Rheological Aging Index, RAI, for the different binders studied.

In Figure 5, the values of the considered Rheological Aging Index, RAI, calculated according to Equation (5), are reported for all the investigated materials.

The values of the aging index provide evidence that the extent of the modifications induced by the RTFOT aging is strongly reduced by the polymer introduction, regardless the type of polymer used for modification.

**Figure 6:**
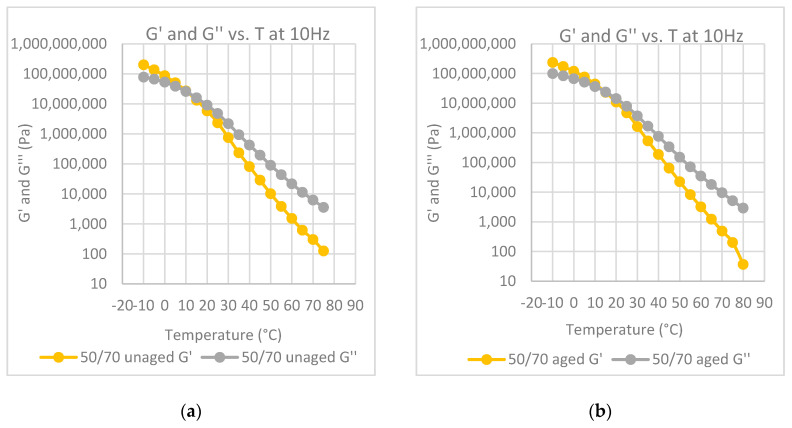

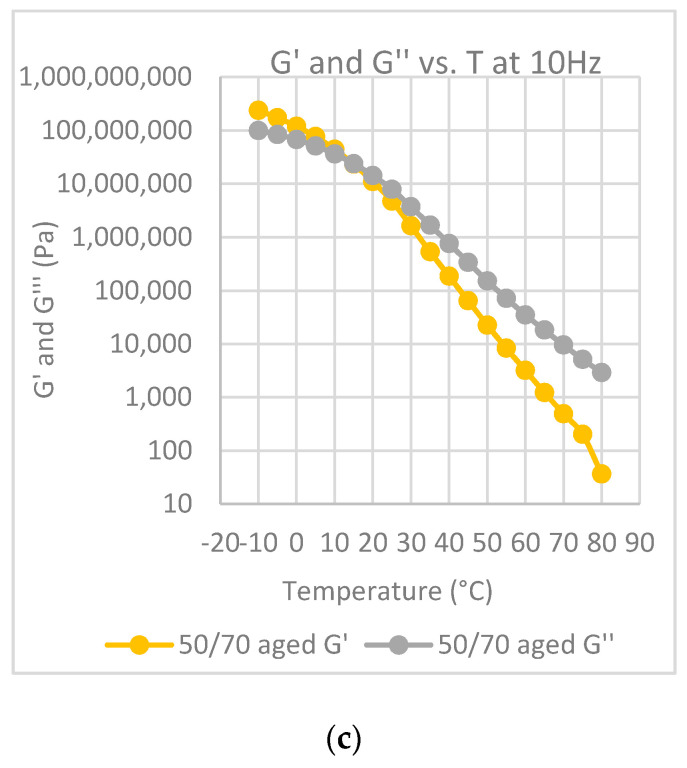
Elastic modulus, G′ and loss modulus, G″, at 10 Hz, as a function of temperature: (**a**) neat bitumen before aging; (**b**) neat bitumen after aging at the RTFOT; (**c**) neat bitumen after mixing protocol at high temperature.

**Figure 7:**
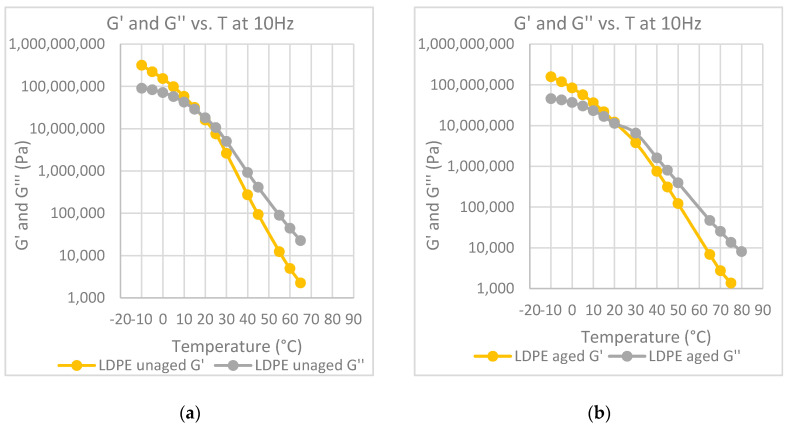
Elastic modulus, G′ and loss modulus, G″, at 10 Hz, as a function of temperature: (**a**) binder modified with LDPE before aging; (**b**) binder modified with LDPE after aging at the RTFOT.

**Figure 8:**
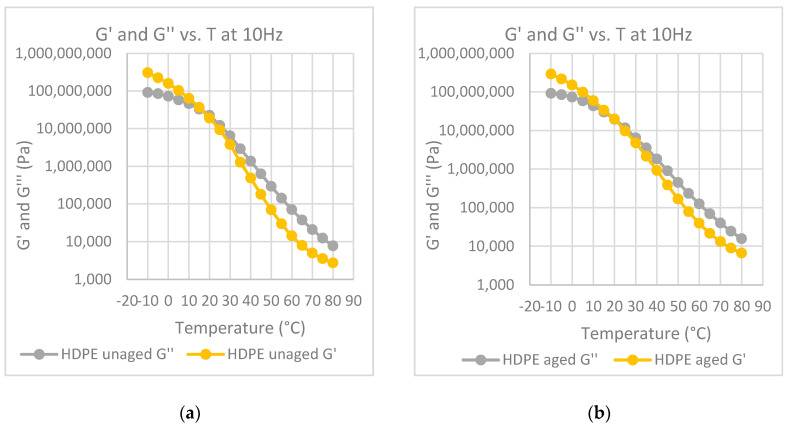
Elastic modulus, G,’ and loss modulus, G″, at 10 Hz, as a function of temperature: (**a**) binder modified with HDPE before aging; (**b**) binder modified with HDPE after aging at the RTFOT.

**Figure 9:**
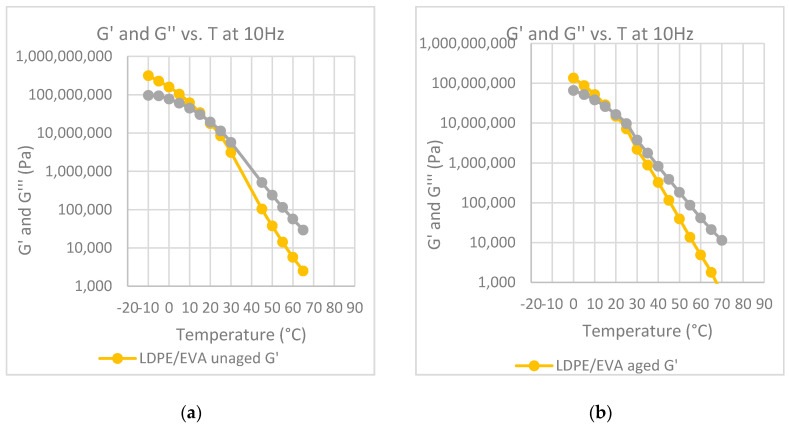
Elastic modulus, G′ and loss modulus, G″, at 10 Hz, as a function of temperature: (**a**) binder modified with LDPE-LLDPE-EVA before aging; (**b**) binder modified LDPE-LLDPE-EVA after aging at the RTFOT.

**Figure 10:**
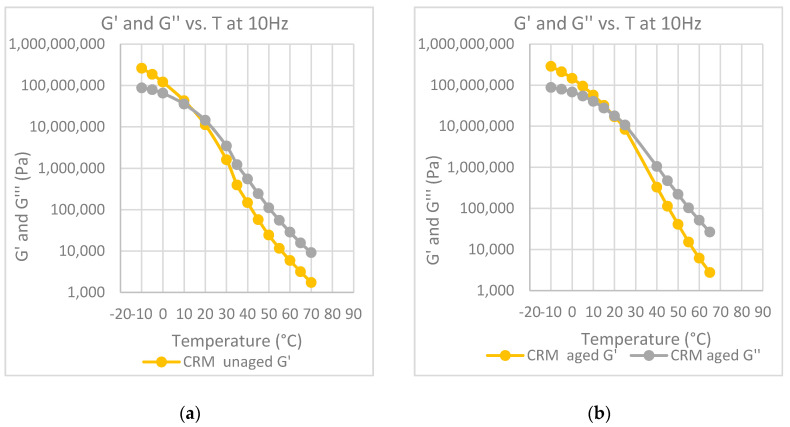
Elastic modulus, G′ and loss modulus, G″, at 10 Hz, as a function of temperature: (**a**) binder modified with CRM before aging; (**b**) binder modified CRM after aging at the RTFOT.

The temperature at which the G′ and G” curves cross each other, the so-called crossover temperature, is reported in Figure 6, Figure 7, Figure 8, Figure 9 and Figure 10 for all the investigated materials. The crossover temperature is the temperature at which the elastic and the viscous components of the materials have the same values, and after increasing the temperature the viscous component becomes predominant. This means that the increase of the crossover temperature remarks an extension of the temperature at which the bitumen behaves as a solid, and then it is an extension of the working temperature.

**Figure 11:**
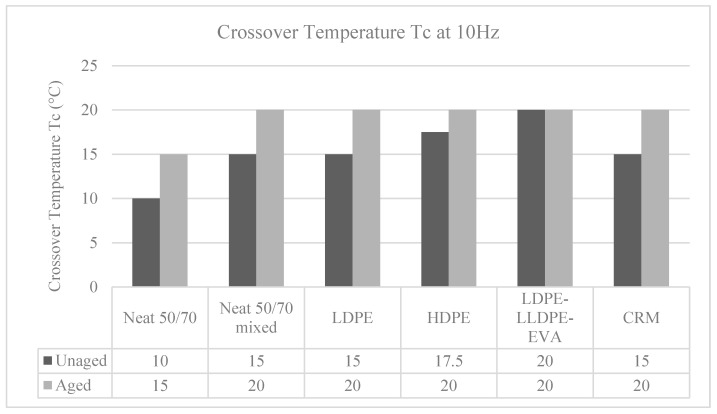
Crossover temperature, Tc, at 10 Hz, for all the binders studied.

The presence of the polymer filler increases the crossover temperature, which increases also after aging, as depicted in Figure 11. Of course, this is a confirmation of the liquid–solid transition of the bitumen and that both the presence of the polymer filler and the aging effect make the mechanical behavior of the bitumen more rigid. Indeed, an excess in rigidity as an excess in reduction of the phase angle [29] may be detrimental for the in situ behavior once in pavement, when considering the needed cracking resistance to repeated traffic loading (fatigue cracking) and thermal changes (thermal cracking).

It is interesting to observe that all the binders that have been modified and subject to thermal treatment (such as the neat binder subject to the mixing protocol), regardless of the type of polymer, have the same crossover temperature, which seems to be a characteristic of the base binder.

### 3.5. Morphology

**Figure 12:**
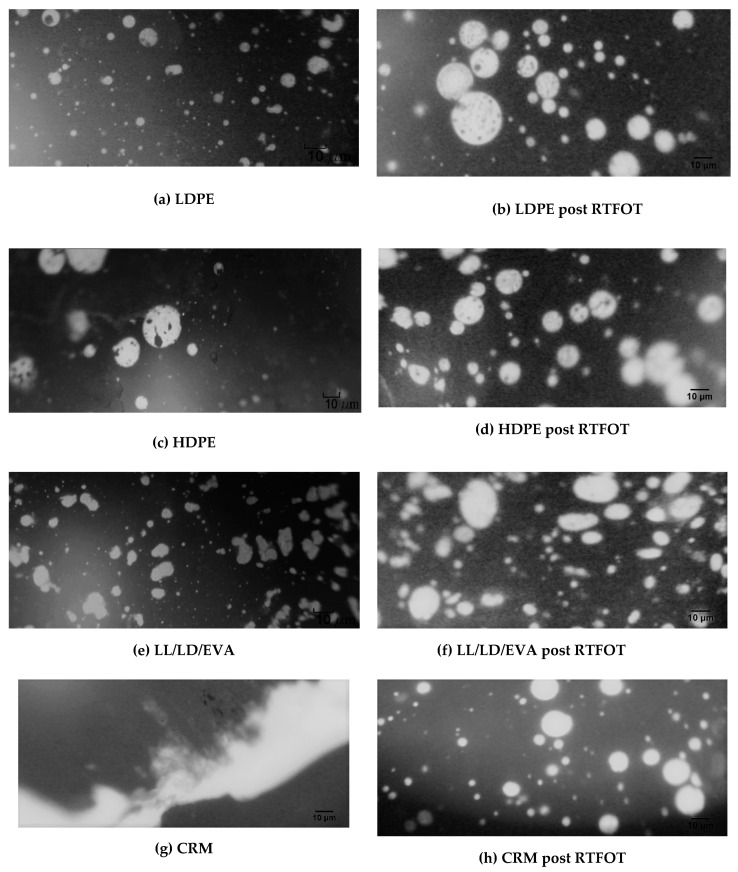
Micrographs of the PMBs produced, before and after short-term aging: (**a**) binder modified with LDPE before aging; (**b**) binder modified with LDPE after aging at the RTFOT; (**c**) binder modified with HDPE before aging; (**d**) binder modified with HDPE after aging at the RTFOT; (**e**) binder modified with LDPE-LLDPE-EVA before aging; (**f**) binder modified with LD/LL/EVA after aging at the RTFOT; (**g**) binder modified with CRM before aging; (**h**) binder modified with CRM after aging at the RTFOT.

Fluorescent microscopy allows identifying the state of dispersion of the polymer within the base bitumen as well as to assess, from a qualitative point of view, the nature of dispersion of the polymer phase (white particles) into the black bituminous phase. Figure 12a,c,e,g show the modified samples before aging, while Figure 12b,d,f,h represent the corresponding samples after short-term aging. This allows a better understanding of the modification induced in the phase structure of the PMBs studies, due to aging [28,33].

Based on the micrographs given in Figure 12, a qualitative description of the PMBs produced was carried out according to the codified procedure described in Table 3, following EN 13632 standard, and results are provided in Table 11.

The pictures confirm a clear change in morphology based on two aspects, the first one is illustrated as the polymer type changes, while the second one is seen when the PMBs are subjected to short-term aging. By changing the type of polymer, each picture shows a respective/representative form of polymer dispersion within the bitumen phase. Before aging, all PMBs have the polymer as disconnected spherical droplets dispersed within the bituminous phase except for CRM, which shows that both phases are continuous by having an elongated polymer matrix within the bitumen medium. Conversely, HDPE is the only polymer to reveal irregular spherical drops that are relatively bigger than the others and irregularly dispersed. This evidence points out a low interface bond between the polymer and bitumen. There is no evidence of strong polymer–bitumen dispersion, and therefore, the network that emphasizes good compatibility between both elements is lacking [33,34].

However, for LDPE and LD/LL/EVA, irregular elongated shapes of polymers more dispersed into the bitumen medium are illustrated: this indicates a blend that possesses more homogeneous dispersion; therefore, it tends to have better compatibility and rheological behavior. After the exposure of all samples to short-term aging, a relevant change in the internal structure of all the PMBs without exception is evident. This highlights that the oxidative aging effect is detectible through morphology analysis where polymers tend to dissolve in a dispersion of thinner and more spread particles. Such polymer distribution of smaller sized particles within the bitumen phase is favorable, indicating that aging improves the diffusion of the polymer within the bitumen. This structural change matches the change in rheological properties as the discussed increase in elastic modulus and viscosity for the PMBs after aging. Short-term aging appears to have a favorable impact on the dispersion of the polymer, as it aids the miscibility of polymers within the bituminous phase. However, this is valid when the properties of the mixture are governed predominantly depending on the polymer dispersion phase, as well as by the type of the polymer [35,36]. Indeed, the degree of modification is related not only to the base bitumen structure, but also to the type of polymer in study.

## 4. Conclusions

Many aging indices have been proposed in the literature for the characterization of PMBs: most of them do not provide coherent results when associated with evidences in terms of the morphology or mechanical performance of these type of materials. Therefore, a better understanding of such evolution is needed in order to improve the testing protocol that is suitable for evaluating the short-term aging of PMBs.

Therefore, an aging index based on the elastic component of the complex modulus, G′, over a wide frequency range has been calculated: the results prove that it provides clear information on the mechanical effect of aging on the binders studied and that the effect of polymer introduction is substantially based on it. In particular, effect of laboratory-induced aging is strongly reduced by the polymer introduction, regardless the type of polymer used for modification, while the crossover temperature after aging and/or thermal stress increases to a constant value that seems to be a characteristic of the base binder that is not related to the modification or to the type of modifier.

Furthermore, the results also prove that the variation in the properties of the bitumen after polymer modification depends—to some extent—not only on the type and content of the polymer added, but also on the thermal stress induced during the production of the PMB, as seen when comparing the results for the PMBs produced and those for the base bitumen subject to high shear mixing at high temperature.

## Figures and Tables

**Table 1:** Conventional properties of the neat bitumen.

Physical Properties	Specific Gravity @25 °C	Penetration	Softening Point	Fraass Breaking Point	Ductility
Units	g/cm^3^	0.1 mm	(°C)	(°C)	(mm)
Specification	EN-ISO-3838	EN 1426	EN 1427	EN 12593	EN 13398
Test results	1.051	68	50.5	−12	over 100
Requirements	-	50–70	46–54	≤−8	≥100

**Table 2:** SARA fraction characteristics of the neat bitumen.

Fraction	Polarity	Percentage (%)	Characteristics	Color
Saturates	Non-polar	2.4	Viscous oil	White
Aromatics	Non-polar	55.6	Viscous liquid	Dark brown
Resins	Highly polar	25.4	Solid to semi-solid	Dark brown
Asphaltenes	Highly polar	16.6	Solid	Brown to black

**Table 3:** Polymers used in this work.

Polymer	Sample Code	MFI, g/10 min	Density (g/cm3)	Melting Point (°C)	Degree of Crystallinity (%)
LDPE,	Riblene FC39	0.25	0.924	114	32
HDPE,	Eraclene MP 90	7	>0.96	137	67
LLDPE	Clearflex FG106	1	0.918	125	-
EVA ^1^	Greenflex ML 60	2.5	-	74	7

^1^ Vinyl acetate content (%) = 28%.

**Table 4:** Crumb rubber characteristics.

Characteristic	Description
Type	Powder and fine granulate
Specific gravity [g/cm^3^]	1.10–1.20
Color	Black
Grinding method	mechanical
source	Rubber granulate from waste tires
Average diameter [mm]	<0.4

**Table 5:** Letter coding description according to EN13632:2005.

Phase continuity	P: continuous polymer phase
	B: Continuous bitumen phase
	X: Continuity of both (crosslinking)
Phase description	H: Homogeneous
	I: Heterogeneous
Size description	S: Small (<10 μm)
	M: Medium (from 10 μm to 100 μm)
	L: Large (>100 μm)
Shape description	r: Round, cylindrical
	s: Elongated
	o: Other

**Table 6:** Conventional tests of polymer-modified binders post rolling thin film oven test (RTFOT).

Requirement	Pen @ 25 °C	T_R&B_	Penetration Index PI	Pen @ 25 °C	T_R&B_	Penetration Index PI	∆T_R&B_	Change in mass
**Characteristics**	**Pre-RTFO**			**Post-RTFOT**				**M**
**Standard**	EN 1426	EN 1427	EN 12591	EN 1426	EN 1427	EN 12591	EN 12607-1	
**Unit**	dmm	°C		dmm	°C		°C	%
**BITUMEN 50/70**	68	50	−0.46	44	54	−0.55	4	−0.19
**LDPE**	35.7	56	−0.57	23	62.25	−0.23	6.25	−0.82
**HDPE**	25.7	58	−0.81	18.4	64.25	−0.29	6.25	−0.87
**LD/LL/EVA**	35	61	0.39	20	63	−0.72	2	−0.47
**CRM**	45	58	0.36	28	63	0.29	5	

**Table 7:** Storage stability and elastic recovery.

Requirements	Storage Stability	Elastic Recovery
Characteristics	∆T_R&B_	Average
Standard	EN 13399	EN 13398
Unit	°C	%
LDPE FC39	28	9.25
HDPE MP90	20	9.5
LD/LL/EVA	5	15
CRM	14	60

**Table 8:** Dynamic viscosity for the binder studied, before and after aging.

Test	Dynamic Viscosity Pre-RTFO				Dynamic Viscosity Post-RTFOT			
Temperature (°C)	180	150	135	100	180	150	135	100
Standard	EN 13702							
unit	Pa∙s							
Bitumen 50/70	-	0.22	0.44	3.92	0.05	0.23	0.47	4.50
Bitumen 50/70 mixed	0.08	0.25	0.52	4.91	0.10	0.32	0.67	7.13
LDPE FC39	0.15	0.46	0.92	8.78	0.18	0.58	1.23	13.30
HDPE MP90	0.15	0.46	0.98	16.30	0.21	0.67	1.43	28.20
LD/LL/EVA	0.17	0.50	1.00	8.28	0.20	0.64	1.34	12.70
CRM	-	0.32	0.59	5.47	0.23	0.70	1.46	15.30

**Table 9:** Parameters of the Arrhenius equation for the binders studied before and after aging.

Stage	Unaged			After Short-Term Aging		
Binder	A	*Ea* (kJ/mol)	R²	A	*Ea* (kJ/mol)	R²
Bitumen 50/70	8 × 10^−11^	72.46	0.9921	5 × 10^−10^	77.79	0.9993
Bitumen 50/70 mixed	3 × 10^−10^	72.88	0.9941	1 × 10^−10^	76.41	0.9939
LDPE	7 × 10^−10^	71.67	0.9921	3 × 10^−10^	76.05	0.9924
HDPE	3 × 10^−11^	82.98	0.9795	2 × 10^−10^	86.76	0.9769
LD/LL/EVA	2 × 10^−9^	69.97	0.9911	6 × 10^−10^	73.82	0.9934
CRM	1 × 10^−10^	75.51	0.9961	5 × 10^−10^	74.76	0.9918

**Table 10:** Aging indices for rotational viscosity, penetration, and ring and ball temperature.

Polymer	AI_RV_				AI_TR&B_	AI_PEN_ (%)
T (°C)	180	150	135	100		
Bitumen 50/70	-	1.02	1.08	1.15	1.08	64.71
Bitumen 50/70 mixed	1.19	1.26	1.29	1.45	-	-
LDPE	1.19	1.25	1.34	1.51	1.11	64.43
HDPE	1.40	1.45	1.46	1.73	1.10	71.6
LD/LL/EVA	1.19	1.30	1.37	1.48	1.03	71.43
CRM	-	2.17	2.46	2.80	1.09	62.22

**Table 11:** Morphological characterization of the PMBs studied.

STAGE	Unaged				After Short-Term Aging			
PMB	Phase continuity	Phase description	Size description	Shape description	Phase continuity	Phase description	Size description	Shape description
LDPE	B	I	S	r/o	B	I	M	r
HDPE	B	I	M	r/o	B	I	M	r/o
LD/LL/EVA	B	H	S	s/r	B	I	M	s/r
CRM	X	I/H	S	r/o	B	I	M	r
